# Evaluation of the prognostic significance of HER family mRNA expression in high-risk early breast cancer: a Hellenic Cooperative Oncology Group (HeCOG) validation study

**DOI:** 10.1186/s12967-015-0530-0

**Published:** 2015-05-29

**Authors:** Angelos Koutras, Konstantine T Kalogeras, Ralph M Wirtz, Zoi Alexopoulou, Mattheos Bobos, Flora Zagouri, Elke Veltrup, Eleni Timotheadou, Helen Gogas, George Pentheroudakis, Nikolaos Pisanidis, Christina Magkou, Christos Christodoulou, Dimitrios Bafaloukos, Pavlos Papakostas, Gerasimos Aravantinos, Dimitrios Pectasides, Haralambos P Kalofonos, George Fountzilas

**Affiliations:** Division of Oncology, Department of Medicine, University Hospital, University of Patras Medical School, 26504 Rio, Greece; Laboratory of Molecular Oncology, Hellenic Foundation for Cancer Research, Aristotle University of Thessaloniki School of Medicine, University Campus, Bldg. 17B, 54 006 Thessaloniki, Greece; Translational Research Section, Hellenic Cooperative Oncology Group, Data Office, 18 Hatzikonstanti Str., 115 24 Athens, Greece; STRATIFYER Molecular Pathology GmbH, Werthmannstr. 1c, 50935 Cologne, Germany; Health Data Specialists Ltd, 22 Katehaki Str., 115 25 Athens, Greece; Department of Clinical Therapeutics, “Alexandra” Hospital, University of Athens School of Medicine, 80 Vasilissis Sofias Av. and Lourou Str., 115 28 Athens, Greece; Department of Medical Oncology, “Papageorgiou” Hospital, Aristotle University of Thessaloniki School of Medicine, Ring Road, Nea Efkarpia, 56 429 Thessaloniki, Greece; First Department of Medicine, “Laiko” General Hospital, University of Athens School of Medicine, 17 Ag. Thoma Str., 115 27 Athens, Greece; Department of Medical Oncology, Ioannina University Hospital, Niarchou Av., 45 500 Ioannina, Greece; Department of Medical Oncology, IKA Hospital, 40 Mavromichali Str., 54248 Thessaloniki, Greece; Pathology Department, “Evangelismos” Hospital, 45 Ipsilantou Str., 106 76 Athens, Greece; Second Department of Medical Oncology, “Metropolitan” Hospital, 9 Ethnarchou Makariou Str. and El. Venizelou Str., 185 47 Piraeus, Greece; First Department of Medical Oncology, “Metropolitan” Hospital, 9 Ethnarchou Makariou Str. and El. Venizelou Str., 185 47 Piraeus, Greece; Oncology Unit, “Hippokration” Hospital, 108 Vas. Sofias Av., 115 27 Athens, Greece; Second Department of Medical Oncology, “Agii Anargiri” Cancer Hospital, Noufaron Str. and Timiou Stavrou Str., 145 64 Athens, Greece; Oncology Section, Second Department of Internal Medicine, “Hippokration” Hospital, 114 Vas. Sofias Av., 115 27 Athens, Greece

**Keywords:** HER family, mRNA, qRT-PCR, Prognostic value, Breast cancer

## Abstract

**Background:**

The aim of the study was to evaluate the prognostic ability of the transcriptional profiling of the HER family genes in early breast cancer, as a validation analysis of another previously published HeCOG study.

**Methods:**

RNA was extracted from 663 formalin-fixed paraffin-embedded (FFPE) tumor tissue samples of high-risk early breast cancer patients enrolled in the randomized HE10/00 trial. Relative mRNA expression of all four HER family members was assessed by quantitative reverse transcription-polymerase chain reaction (qRT-PCR).

**Results:**

In compliance with our previous study, the overall agreement between qRT-PCR and IHC/FISH for HER2 status determination was good (69%). Likewise, the overall concordance between qRT-PCR and IHC for EGFR status was high (81%). In line with our previously reported data, we demonstrated a positive association between HER2 and HER3 mRNA expression. Similarly, mRNA expression of HER3 and HER4 was positively associated with each other and negatively associated with EGFR. Regarding relationships with clinico-pathological parameters, our findings are also in agreement with our previous results. Generally, increased EGFR and HER2 mRNA expression was related to unfavorable, whereas high HER3 and HER4 mRNA expression was associated with favorable clinico-pathological parameters. In univariate analysis, no significant association between EGFR, HER2 and HER3 mRNA expression and overall survival (OS) or disease-free survival (DFS) was demonstrated. However, high EGFR protein expression was associated with significantly shorter OS (log-rank, p = 0.015). In compliance with our previously published data, increased HER4 mRNA expression had a significantly favorable prognostic value in terms of OS (p = 0.044) and DFS (p = 0.047). In multivariate analysis, among all HER receptors, only EGFR protein expression was found to affect OS (Wald’s p = 0.028) and DFS (p = 0.015) independently. Concerning the combined expression of all four HER family receptors, the combination of high EGFR, high HER2, low HER3 and low HER4 mRNA expression was associated with a trend for shorter OS (log-rank, p = 0.065) and significantly worse DFS (p = 0.033), compared with all other co-expression profiles.

**Conclusions:**

These data indicate that qRT-PCR may represent a valid alternative method for evaluating the expression of HER family members in FFPE breast carcinoma tissue samples.

**Trial registration:**

Australian New Zealand Clinical Trials Registry ACTRN12609001036202

## Background

The human epidermal growth factor receptor (HER) family consists of four homologous members: ErbB-1 (epidermal growth factor receptor [EGFR] or HER1), ErbB-2 (HER2), ErbB-3 (HER3) and ErbB-4 (HER4) [1]. The HER family constitutes a promising area for the development of targeted treatments in patients with breast cancer and considerable therapeutic progress has already been achieved. Apart from HER2, the significance of lateral signaling partners is also increasingly recognized, given the role of dimerization among HER receptors. Therefore, the evaluation of all HER family members as a whole is considered important.

Overexpression and/or amplification of the HER2 receptor occurs in 15–30% of breast cancer cases and is associated with aggressive course of the disease and unfavorable clinical outcome [2]. HER2 status can be evaluated at the DNA, the mRNA or the protein level. Although various assays are available, immunohistochemistry (IHC) is the technique of choice in the routine practice. Fluorescence in situ hybridization (FISH) analysis is required in cases with (2+) HER2 immunostaining [3]. However, despite efforts to standardize these techniques, there is a considerable intra- and inter-laboratory variability of the results [4, 5]. Quantitative reverse transcription-polymerase chain reaction (qRT-PCR) represents an alternative test for the determination of HER2 status. qRT-PCR does not necessitate experienced pathologists to interpret the results and is associated with reproducible and quantitative findings. Previous reports have demonstrated that the qRT-PCR can be applied in archival formalin-fixed paraffin-embedded (FFPE) tissues [6].

In a previous study [7], we investigated the potential prognostic value of the transcriptional profiling of all four HER family genes in patients with high-risk early breast cancer, receiving dose-dense anthracycline-based sequential adjuvant chemotherapy with or without paclitaxel. The results of this analysis, using qRT-PCR, suggested that EGFR and HER2 are unfavorable prognostic markers, while HER3 and HER4 mRNA expression is related to better clinical outcome. In order to validate the above findings in the current study, we evaluated the prognostic ability of HER family mRNA expression using qRT-PCR, in a larger series of high-risk patients with early breast cancer. These patients were treated with dose-dense sequential or concurrent epirubicin and paclitaxel, followed by ‘intensified’ CMF, within the context of the Hellenic Cooperative Oncology Group (HeCOG) 10/00 randomized phase III trial [8].

## Methods

### Clinical study

The HE10/00 trial [8] was a randomized phase III study (ACTRN12609001036202). Patients were treated with three cycles of epirubicin (E), followed by three cycles of paclitaxel (T, Taxol, Bristol Myers-Squibb, Princeton, NJ), followed by three cycles of intensified CMF (cyclophosphamide, methotrexate and 5-fluorouracil) (E–T–CMF, all cycles given every 2 weeks) or with four cycles of epirubicin/paclitaxel (ET) combination (given on the same day every 3 weeks) followed by three cycles of intensified CMF every 2 weeks (ET–CMF). By study design, the cumulative doses and the chemotherapy duration were identical in the two arms but dose intensity of epirubicin and paclitaxel was double in the E–T–CMF arm. A total of 1,086 eligible patients with node-positive operable breast cancer were accrued in a period of 5 years (2000–2005). HER2-positive patients received trastuzumab upon relapse, as previously described [9]. Treatment schedules, baseline characteristics and clinical outcomes have already been described in detail [8]. Primary tumor diameter, axillary nodal status and tumor grade were obtained from the pathology report. The clinical protocol was approved by local regulatory authorities, while the present translational research studies were approved by the Bioethics Committee of the Aristotle University of Thessaloniki School of Medicine. All patients signed a study-specific written informed consent before randomization, which in addition to giving consent for the trial allowed the use of biological material for future research purposes.

### Tissue microarray (TMA) construction

**Figure 1:**
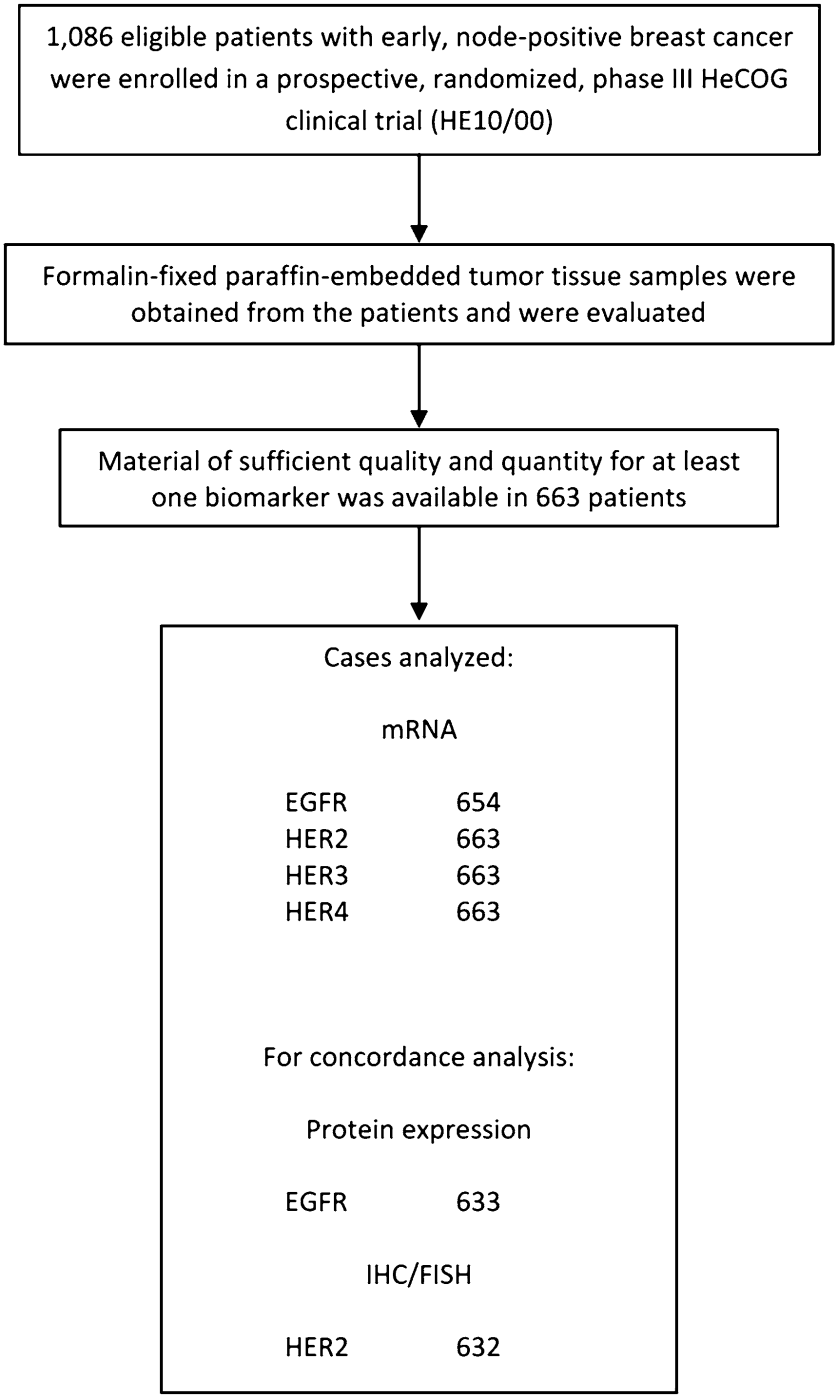
REMARK diagram.

Formalin-fixed paraffin-embedded (FFPE) tumor tissue samples were prospectively collected from 663 patients (61% of 1,086 randomized patients) who were part of the HE10/00 study population. For 39% of the randomized patients, we were either not successful in obtaining an FFPE tumor tissue sample or the sample obtained was not of adequate quality or quantity for biomarker evaluation. The REMARK diagram [10] for the study is shown in Figure 1. Hematoxylin–eosin stained sections from the tissue blocks were reviewed by two experienced breast cancer pathologists and the most representative tumor areas were marked for the construction of the ΤΜΑ blocks with the use of a manual arrayer (Model I, Beecher Instruments, San Prairie, WI), as previously described [11, 12]. Each case was represented by two tissue cores, 1.5 mm in diameter, obtained from the most representative areas of primary invasive tumors or in some cases (7.2%) from synchronous axillary lymph node metastases and re-embedded in 35 microarray blocks. Each TMA block contained 38–66 tissue cores from the original tumor tissue blocks, while cores from various neoplastic, non-neoplastic and reactive tissues were also included, serving as controls for slide-based assays. Cases not represented, damaged or inadequate on the TMA sections were re-cut from the original blocks and these sections were used for protein and gene analysis. Histological grade was evaluated according to the Scarff, Bloom and Richardson system.

### Immunohistochemistry (IHC)

Immunohistochemical labeling was performed according to standard protocols on serial 2.5 μm thick sections from the TMA blocks or the original blocks. All cases were also stained for vimentin (clone V9, Dako, Glostrup, Denmark) and cytokeratin 8/18 (clone 5D3, Novocastra™, Leica Biosystems, Newcastle, UK), which were used as control stains for tissue immunoreactivity and fixation, as well as identification of tumor cells. Tissue samples negative for the above antibodies were excluded from the study. To assure optimal reactivity, immunostaining was done 7–10 days after sectioning at the Laboratory of Molecular Oncology of the Hellenic Foundation for Cancer Research, Aristotle University of Thessaloniki School of Medicine. The staining procedures for EGFR (clone 31G7, Invitrogen, Carlsbad, CA), HER2 (A0485 polyclonal antibody, Dako), estrogen receptor (ER, clone 6F11, Novocastra™, Leica Biosystems), progesterone receptor (PgR, clone 1A6, Novocastra™, Leica Biosystems) and Ki67 (clone MIB-1, Dako) were performed using a Bond Max™ autostainer (Leica Microsystems, Wetzlar, Germany), as previously described [13].

### Interpretation of the IHC results

The evaluation of all IHC sections was done by two experienced breast cancer pathologists, blinded as to the patients’ clinical characteristics and survival data, according to existing established criteria, as previously described [9]. Briefly, EGFR protein expression was scored in a scale from 0 to 3+ and was considered to be positive if membraneous staining of 1+, 2+ or 3+ was present in ≥1% of tumor cells [14]; HER2 protein expression was scored in a scale from 0 to 3+, the latter corresponding to uniform, intense membrane staining in >30% of invasive tumor cells [15]; ER and PgR were evaluated using the Histoscore method (max score 400) and were considered positive if staining was present in ≥1% of tumor cell nuclei [16] and, for Ki67, the expression was defined as low (<14%) or high (≥14%) based on the percentage of stained tumor cell nuclei [17]. The mean percentage of stained cells from the two cores was calculated, while in cases with different intensities, the higher intensity score obtained from the two cores was used. If one of the tissue cores was lost or damaged the overall score was determined from the remaining one. When whole tissue sections were used, the entire tumor area was evaluated.

### Fluorescence in situ hybridization (FISH)

TMA sections or whole sections (5 μm thick) were cut for FISH analysis, using the ZytoLight^®^ SPEC *HER2*/*TOP2A*/CEP17 triple-color probe kit (ZytoVision, Bremerhaven, Germany). The FISH was performed according to the manufacturer’s protocol with minor modifications. Four carcinoma cell lines (MDA-MB-231, MDA-MB-175, MDA-MB-453 and SK-BR-3) from the Oracle HER2 Control Slide (Leica Biosystems), with a known *HER2* gene status, were also used as a control for the FISH assays and analyzed for *HER2* genomic status.

For all probes, sequential (5 planes at 1.0 μm) digital images were captured using the Plan Apo VC 100×/1.40 oil objective (Nikon, Kanagawa, Japan), using specific filters for each probe. The resulting images were reconstructed using specifically developed software for cytogenetics (XCyto-Gen, ALPHELYS, Plaisir, France). Processed sections were considered eligible for FISH evaluation according to the ASCO/CAP criteria [15]. For the evaluation of *HER2*/*TOP2A*/CEP17 status, non-overlapping nuclei from the invasive part of the tumor were randomly selected, according to morphological criteria using DAPI staining, and scored. The virtual slides of HER2, ER or PgR stains, created as previously described [13] were used for selecting the invasive part of the tumor in each TMA. Twenty tumor nuclei were counted according to Press et al. [18]. The *HER2* gene was considered to be amplified when the *HER2*/CEP17 ratio was ≥2.2 [15], or the mean *HER2* copy number was >6 [19].

In cases with ratios at or near the cut-off (1.8–2.2), additional 20 or 40 nuclei were counted and the ratio was recalculated. In cases with a borderline ratio at 60 nuclei, additional FISH assays were performed in whole sections [20]. All primary image data of the TMA and whole tumor sections have been digitally scanned and made publicly available at: http://www.hecog-images.gr/HER2/TOP2A/CEN17/FISH_HE10/97_HE10/00.

### RNA isolation from FFPE tissue and quantitative reverse transcription-polymerase chain reaction (qRT-PCR) assessment

Prior to RNA isolation, macrodissection of tumor areas was performed in most of the FFPE sections with <50% tumor cell content. More than one FFPE section (10 μm thick) was used for RNA extraction when the tumor surface of a given sample was less than 0.25 cm^2^. From each FFPE section or macrodissected tissue fragments, RNA was extracted using a standardized fully automated isolation method for total RNA from FFPE tissue, based on germanium-coated magnetic beads (XTRAKT kit, STRATIFYER Molecular Pathology GmbH, Cologne, Germany) in combination with a liquid handling robot (XTRAKT XL, STRATIFYER Molecular Pathology GmbH), as previously described [21]. The method involves extraction-integrated deparaffinization and DNase I digestion steps. The quality and quantity of RNA was checked by measuring CALM2 expression as a surrogate for amplifiable mRNA by qRT-PCR. CALM2 was used as endogenous reference, since it had previously been identified as being highly expressed among breast cancer tissue samples.

qRT-PCR primers and labeled hydrolysis probes were selected using Primer Express^®^ Software, Version 2.2 and 3 (Applied Biosystems/Life Technologies, Karlsruhe, Germany), according to the manufacturer’s instructions, and were controlled for single nucleotide polymorphisms. All primers, probes and amplicons were checked for their specificity against nucleotide databases at NCBI using basic local alignment search tool (BLAST). Primers and probes were purchased from Eurogentec S.A. (Seraing, Belgium). For each primer/probe set, the amplification efficiency was tested, aiming to reach comparable efficiency of >90% (efficiency range from 91 to 100%). Primers and hydrolysis probes were diluted to 100 µM, using a stock solution with nuclease-free water (Life Technologies GmbH, Darmstadt, Germany). qRT-PCR was applied for the relative quantification (RQ) of the HER family genes EGFR, HER2, HER3 and HER4. The Primer/probe (FAM/TAMRA-labeled) sets used for amplification of the target and reference genes are shown in Table 1.

**Table 1:** Primer and probe sequences used for quantitative reverse transcription-polymerase chain reaction (qRT-PCR)

Gene symbol	NM_number	Probe name	Probe sequence	Forward name	Forward sequence	Reverse name	Reverse sequence
EGFR	NM_201283	MP483	CCTTGCCGCAAAGTGTGTAACGGAAT	MP483_For	CGCAAGTGTAAGAAGTGCGAA	MP483_Rev	CGTAGCATTTATGGAGAGTGAGTCT
H2N (HER2)	M11730_gen	MP452	AGGCCAAGTCCGCAGAAGCCCT	MP452_For	TCTGGACGTGCCAGTGTGAA	MP452_Rev	CCTGCTCCCTGAGGACACAT
HER3	NM_001982	MP489	CTCAAAGGTACTCCCTCCTCCCGGG	MP489_For	CGGTTATGTCATGCCAGATACAC	MP489_Rev	GAACTGAGACCCACTGAAGAAAGG
HER4	NM_005235	MP086	CACAGACTGCTTTGCCTGCATGAATTTC	MP086_For	GAGGCTGCTCAGGACCTAAGG	MP086_Rev	GAGTAACACATGCTCCACTGTCATT
CALM2	NM_001743	MP501	TCGCGTCTCGGAAACCGGTAGC	MP501_For	GAGCGAGCTGAGTGGTTGTG	MP501_Rev	AGTCAGTTGGTCAGCCATGCT

For PCR, 0.5 µM of each primer and 0.25 µM of each probe were used. All quantitative reverse-transcription PCRs were performed in triplicates using the SuperScript^®^ III Platinum^®^ One-Step qRT-PCR kit (Invitrogen/Life Technologies, Darmstadt, Germany) according to the manufacturer’s instructions. Experiments were performed on a Stratagene Mx3005p (Agilent Technologies, Waldbronn, Germany) with 30 min at 50°C and 2 min at 95°C followed by 40 cycles of 15 s at 95°C and 30 s at 60°C. The lengths of the amplicons detected by the EGFR, HER2, HER3, HER4 and CALM2 assays were 93, 61, 81, 75 and 72 bp, respectively, with PCR efficiencies [E = 1^(10 − slope)^] of 89.1, 97.2, 96.0, 92.0 and 99.7%, respectively. Samples were considered eligible for further investigation when the cycle threshold (CT) values of the housekeeping gene were <32 (triplicate mean values). Relative expression levels (relative quantification, RQ) of the target transcripts were calculated as 40—DCT values (DCT = mean CT target gene − mean CT housekeeping gene) to yield positively correlated numbers and to facilitate comparisons. A commercially available human reference RNA (Stratagene qPCR Human Reference Total RNA, Agilent Technologies, Waldbronn, Germany) was used as positive control. No-template controls were assessed in parallel to exclude contamination.

### Statistical analysis

Continuous data are presented as medians and corresponding ranges, while categorical data are presented as counts and percentages. Separation of continuous markers into high and low expression was performed using predetermined cut-offs based on a previously published study [7]. These cut-offs were the 75th percentile for EGFR and the 50th percentile, or the median as it is usually referred to, for HER2, HER3 and HER4. The Spearman’s correlation coefficient method was used to assess correlations among continuous variables.

Comparisons of categorical with continuous variables were made using the Mann–Whitney or the Kruskal–Wallis tests, while the Chi square test was used for testing associations between categorical variables. Cohen’s Kappa and calculation of sensitivity and specificity were used as assessing tools in order to determine the concordance between the qRT-PCR and IHC/FISH methods.

Disease-free survival (DFS) was measured from the date of diagnosis until verified disease progression, death or last contact (whichever occurred first), while overall survival (OS) was measured from the date of diagnosis until death from any cause or date of last contact. Time-to-event distributions were estimated using Kaplan–Meier curves, while log-rank tests and univariate Cox analyses were used for assessing differences statistically. Possible prognostic significance of the markers among breast tumor subtypes was assessed using univariate Cox analyses with interactions. Univariate tests were determined at the level of 0.1%, controlling for multiple comparisons and keeping the overall type I error rate at the level of 5%. Due to the exploratory nature of this study, p values less than 0.05 were presented in the results section.

In the multivariate Cox regression analysis, significance was determined at the level of 15%, while variable selection was performed based on the likelihood ratio test, among the following factors: number of positive nodes, tumor size, type of surgery, adjuvant radiotherapy, adjuvant hormonal therapy and subtype classification. All tests were two-sided. The statistical analysis complied with the reporting recommendations for tumor marker prognostic studies (REMARK) [10] and was performed using the SAS software (SAS for Windows, version 9.3, SAS Institute Inc., Cary, NC).

## Results

### Study population

Basic patient and tumor characteristics according to treatment arm and in the entire study population are shown in Table 2. The differences among these characteristics between the cohort of 663 patients included in the analysis and the remaining 423 patients of the HE10/00 study that were not included are shown in Table 3. The cohort of patients used in the current analysis included cases with more aggressive characteristics, such as higher histological grade, higher number of positive nodes and tumor size, with higher histological grade being found to be associated with HER2 status (66% in HER2-positive vs. 46% in HER2-negative patients, p < 0.001).

**Table 2:** Basic patient and tumor characteristics according to treatment arm and in the entire study population

	Treatment arm		
	E–T–CMF	ET–CMF	Total
Patients			
N	324	339	663
Age in years			
Mean (SD)	54.2 (11.1)	53.9 (11.3)	54.0 (11.2)
Min–Max	29–79	22–76	22–79

	N (%)	N (%)	N (%)
Adjuvant hormonal therapy			
Yes	234 (74.3)	252 (77.3)	486 (75.8)
No	81 (25.7)	74 (22.7)	155 (24.2)
Not reported	9 (2.8)	13 (3.8)	22 (3.3)
Adjuvant radiotherapy			
Yes	220 (71.0)	260 (80.0)	480 (75.6)
No	90 (29.0)	65 (20.0)	155 (24.4)
Not reported	14 (4.3)	14 (4.1)	28 (4.2)
Age			
<50	117 (36.1)	123 (36.2)	240 (36.2)
≥50	207 (63.9)	216 (63.8)	423 (63.8)
EGFR protein expression			
Positive	50 (15.9)	46 (14.4)	96 (15.2)
Negative (<1%)	264 (84.1)	273 (85.6)	537 (84.8)
Not reported	10 (3.1)	20 (5.9)	30 (4.5)
ER/PgR status			
Negative	84 (26.0)	85 (25.1)	169 (25.5)
Positive	239 (74.0)	254 (74.9)	493 (74.5)
Not reported	1 (0.3)		1 (0.2)
Histological grade			
I–II	154 (47.5)	172 (50.8)	326 (49.2)
III-undifferentiated	170 (52.5)	167 (49.2)	337 (50.8)
HER2 protein expression			
Overexpression	92 (29.3)	103 (31.5)	195 (30.4)
No overexpression	222 (70.7)	224 (68.5)	446 (69.6)
Not reported	10 (3.1)	12 (3.5)	22 (3.3)
HER2 status (IHC/FISH)			
Negative	241 (77.5)	248 (77.3)	489 (77.4)
Positive	70 (22.5)	73 (22.7)	143 (22.6)
Not reported	13 (4.0)	18 (5.3)	31 (4.7)
Histological type			
Invasive ductal	261 (80.6)	268 (79.1)	529 (79.8)
Invasive lobular	27 (8.3)	37 (10.9)	64 (9.6)
Mixed	22 (6.8)	19 (5.6)	41 (6.2)
Other	14 (4.3)	15 (4.4)	29 (4.4)
Interval from operation			
<2 weeks	27 (8.3)	22 (6.5)	49 (7.4)
2–4 weeks	144 (44.6)	147 (43.5)	291 (44.0)
>4 weeks	152 (47.1)	169 (50.0)	321 (48.6)
Not reported	1 (0.3)	1 (0.3)	2 (0.3)
Menopausal status			
Pre	137 (42.3)	145 (42.8)	282 (42.6)
Post	187 (57.7)	194 (57.2)	381 (57.4)
Number of positive nodes			
1–3	143 (44.1)	159 (46.9)	302 (45.6)
≥4	181 (55.9)	180 (53.1)	361 (54.4)
Tumor size			
≤2 cm	90 (27.8)	107 (31.6)	197 (29.8)
>2 cm	234 (72.2)	232 (68.4)	466 (70.2)
Subtype classification			
Luminal A	93 (30.5)	97 (30.3)	190 (30.4)
Luminal B	105 (34.4)	112 (35.0)	217 (34.7)
Luminal-HER2	35 (11.5)	44 (13.7)	79 (12.6)
HER2-enriched	35 (11.5)	29 (9.1)	64 (10.3)
Triple-negative	37 (12.1)	38 (11.9)	75 (12.0)
Not reported	19 (5.9)	19 (5.6)	38 (5.7)
Type of surgery			
Modified radical mastectomy	219 (67.6)	227 (67.0)	446 (67.3)
Breast-conserving	105 (32.4)	112 (33.0)	217 (32.7)

Patient characteristics were well balanced between the two arms.

**Table 3:** Basic patient and tumor characteristics in cases included and not included in the analysis

Entire HE10/00 cohort: 1,086 patients	Included (N = 663)	Not included (N = 423)	p value
	N (%)	N (%)	
Adjuvant hormonal therapy			
Yes	486 (75.8)	293 (73.3)	0.35
No	155 (24.2)	107 (26.7)	
Adjuvant radiotherapy			
Yes	480 (75.6)	296 (73.8)	0.52
No	155 (24.4)	105 (26.2)	
Age			
<50	240 (36.2)	195 (46.2)	**0.001**
≥50	423 (63.8)	227 (53.8)	
EGFR protein expression			
Positive	96 (15.2)	22 (17.5)	0.52
Negative (<1%)	537 (84.8)	104 (82.5)	
ER/PgR status			
Negative	169 (25.5)	116 (27.5)	0.48
Positive	493 (74.5)	306 (72.5)	
Histological grade			
I–II	326 (49.2)	237 (56.3)	**0.022**
III-undifferentiated	337 (50.8)	184 (43.7)	
HER2 protein expression			
Overexpression	195 (30.4)	165 (40.6)	**0.001**
No overexpression	446 (69.6)	241 (59.4)	
HER2 status (IHC/FISH)			
Negative	489 (77.4)	98 (73.1)	0.29
Positive	143 (22.6)	36 (26.9)	
Histological type			
Invasive ductal	529 (79.7)	323 (76.5)	0.18
Invasive lobular	64 (9.7)	45 (10.7)	
Mixed	41 (6.2)	23 (5.5)	
Other	29 (4.4)	31 (7.3)	
Interval from operation			
<2 weeks	49 (7.4)	33 (7.9)	0.79
2–4 weeks	291 (44.0)	192 (45.7)	
>4 weeks	321 (48.6)	195 (46.4)	
Menopausal status			
Pre	282 (42.5)	220 (52.0)	**0.002**
Post	381 (57.5)	203 (48.0)	
Number of positive nodes			
1–3	302 (45.6)	224 (53.0)	**0.017**
≥4	361 (54.4)	199 (47.0)	
Tumor size			
≤2 cm	197 (29.7)	149 (35.5)	**0.048**
>2 cm	466 (70.3)	271 (64.5)	
Subtype classification			
Luminal A	190 (30.4)	34 (27.6)	0.40
Luminal B	217 (34.7)	35 (28.5)	
Luminal-HER2	79 (12.7)	21 (17.1)	
HER2-enriched	64 (10.2)	14 (11.4)	
Triple-negative	75 (12.0)	19 (15.4)	
Type of surgery			
Modified radical mastectomy	446 (67.3)	262 (62.1)	0.080
Breast-conserving	217 (32.7)	160 (37.9)	

Significant p values are shown in bold.

### Relative mRNA expression values of HER family receptors

**Figure 2:**
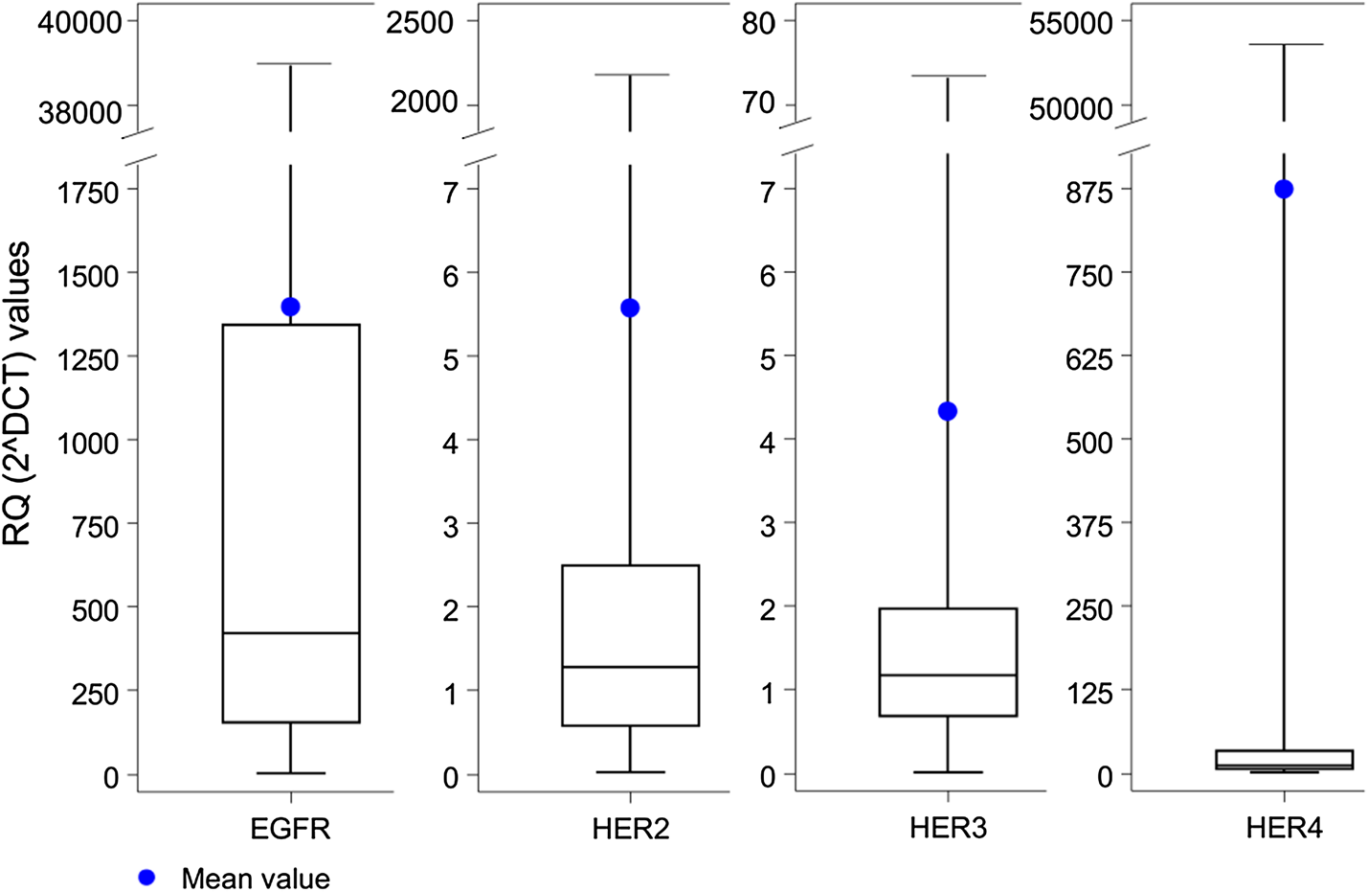
Distribution of relative expression values of mRNA encoding for HER family receptors. The 2 to the power of DCT method was used for data calculation. *CT* cycle threshold, *DCT* delta CT, *RQ* relative quantification.

The distribution of relative expression values (2 to the power of DCT) of mRNA encoding for HER family members is shown in Figure 2. The median value for EGFR was 421.7 (range 1–38,431), for HER2 1.3 (range 0.02–2,256), for HER3 1.2 (range 0.01–73.1) and for HER4 10.7 (range 0.12–53,231).

### Concordance between qRT-PCR and IHC

The total number of samples with data available from both IHC/FISH and qRT-PCR was 625 and 632 for EGFR and HER2, respectively. For EGFR, 93 of the 625 tumors (14.9%) were IHC positive, whereas 153 tumors (24.5%) had EGFR mRNA expression at or above the 75th percentile, as assessed by qRT-PCR. For HER2, 143 of the 632 tumors (22.6%) were IHC/FISH positive, whereas 316 tumors (50.0%) had HER2 mRNA expression at or above the median, as assessed by qRT-PCR. For these tumors, we found a statistically significant association between the evaluations obtained by the two methods, for the EGFR (Chi square test, p < 0.001) and the HER2 (Chi square test, p < 0.001) receptors. The observed overall concordance between the determination of HER2 by qRT-PCR and IHC/FISH was 69.1%. The levels for sensitivity and specificity were 92.3 and 62.4%, respectively, while Cohen’s kappa was 0.38 (95% CI 0.32–0.44). The overall agreement between qRT-PCR and IHC for EGFR was 80.8%. Sensitivity and specificity were 67.7 and 83.1%, respectively, while Cohen’s kappa was 0.40 (95% CI 0.32–0.49) (Table 4).

**Table 4:** Comparison of EGFR and HER2 mRNA expression with EGFR protein expression and HER2 status, respectively

EGFR mRNA (cut-off at 75%)	EGFR protein expression (N = 625)		NPV/PPV (%)	Sensitivity (%)	Specificity (%)
	Negative	Positive			
Low	442 (83.1%)	30 (32.3%)	93.6/41.2	67.7	83.1
High	90 (16.9%)	63 (67.7%)			

HER2 mRNA (cut-off at 50%)	HER2 (IHC/FISH) (N = 632)		NPV/PPV (%)	Sensitivity (%)	Specificity (%)
	Negative	Positive			
Low	305 (62.4%)	11 (7.7%)	96.5/41.8	92.3	62.4
High	184 (37.6%)	132 (92.3%)			

*NPV* negative predictive value, *PPV* positive predictive value.

### Relationships among HER family receptor mRNA expression

**Figure 3:**
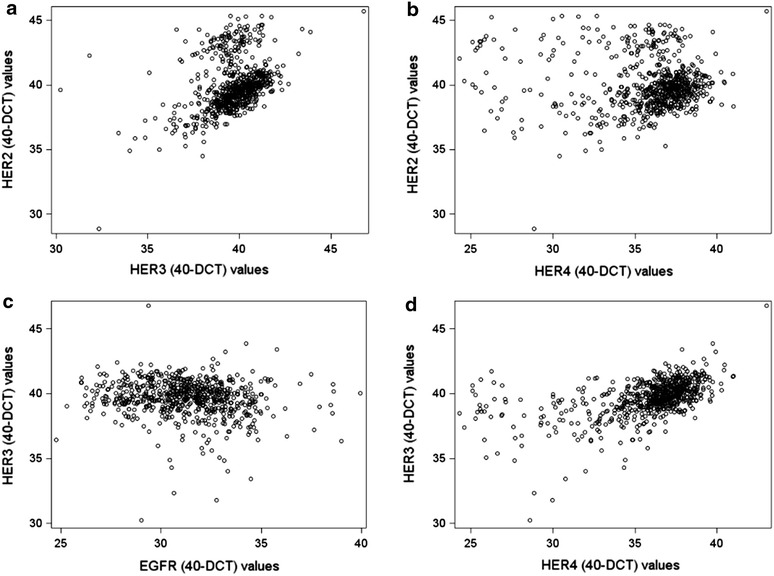
*Scattered plots* of mRNA expression values (40-DCT) of different HER family members. **a** HER2 vs. HER3; **b** HER2 vs. HER4; **c** HER3 vs. EGFR; and **d** HER3 vs. HER4. *CT* cycle threshold, *DCT* delta CT.

A positive correlation was demonstrated between HER2 and HER3 mRNA expression levels (r = 0.42, p < 0.0001), as well as between HER2 and HER4 mRNA values (r = 0.11, p = 0.007). Furthermore, HER3 and HER4 mRNA expression levels were positively correlated to each other (r = 0.56, p < 0.0001) and negatively so with EGFR (r = −0.16, p < 0.0001 and r = −0.15, p < 0.0001, respectively). Scattered plots of the mRNA expression values of different HER family members are presented in Figure 3.

### Association of HER family receptor mRNA expression with clinicopathological parameters

EGFR mRNA expression was inversely associated with the presence of ER (Mann–Whitney, p < 0.001) and PgR (p = 0.005). Moreover, EGFR mRNA expression was inversely related to age (p = 0.003), post-menopausal status (p = 0.003) and tumor size (p = 0.001). A negative correlation between HER2 mRNA expression and PgR was found (p = 0.003). HER3 mRNA expression was associated with ER (p < 0.001) and PgR positivity (p < 0.001), as well as with histological grade I/II (p = 0.001). In addition, HER3 mRNA expression was inversely related to the number of positive lymph nodes (p = 0.017). HER4 mRNA expression was correlated with ER and PgR positivity (p < 0.001 and p < 0.001, respectively), as well as with histological grade I/II (Kruskal–Wallis, p < 0.001). Furthermore, HER4 was associated with lobular and mixed histology (p = 0.002).

Regarding the associations of HER family receptor mRNA expression with breast cancer subtypes, high EGFR mRNA expression was associated with triple-negative breast cancer (TNBC) (p < 0.001), while high HER2 mRNA expression with luminal-HER2 and HER2-enriched subtypes (both, p < 0.001). High HER3 mRNA expression was associated with luminal A and luminal B subtypes (both, p < 0.001), whereas high HER4 mRNA expression with luminal A tumors (p < 0.001).

### Prognostic value of HER family receptor mRNA expression

Survival status of the patients was updated in March 2012. The median follow-up time was 98.9 months (range 0.1–132.5 months). During this time, 218 patients (32.9%) had developed a relapse and 151 patients (22.8%) had died. The 8-year OS was 78.2%, whereas the 8-year DFS was 68.5%. The median OS and DFS have not been reached yet.

**Figure 4:**
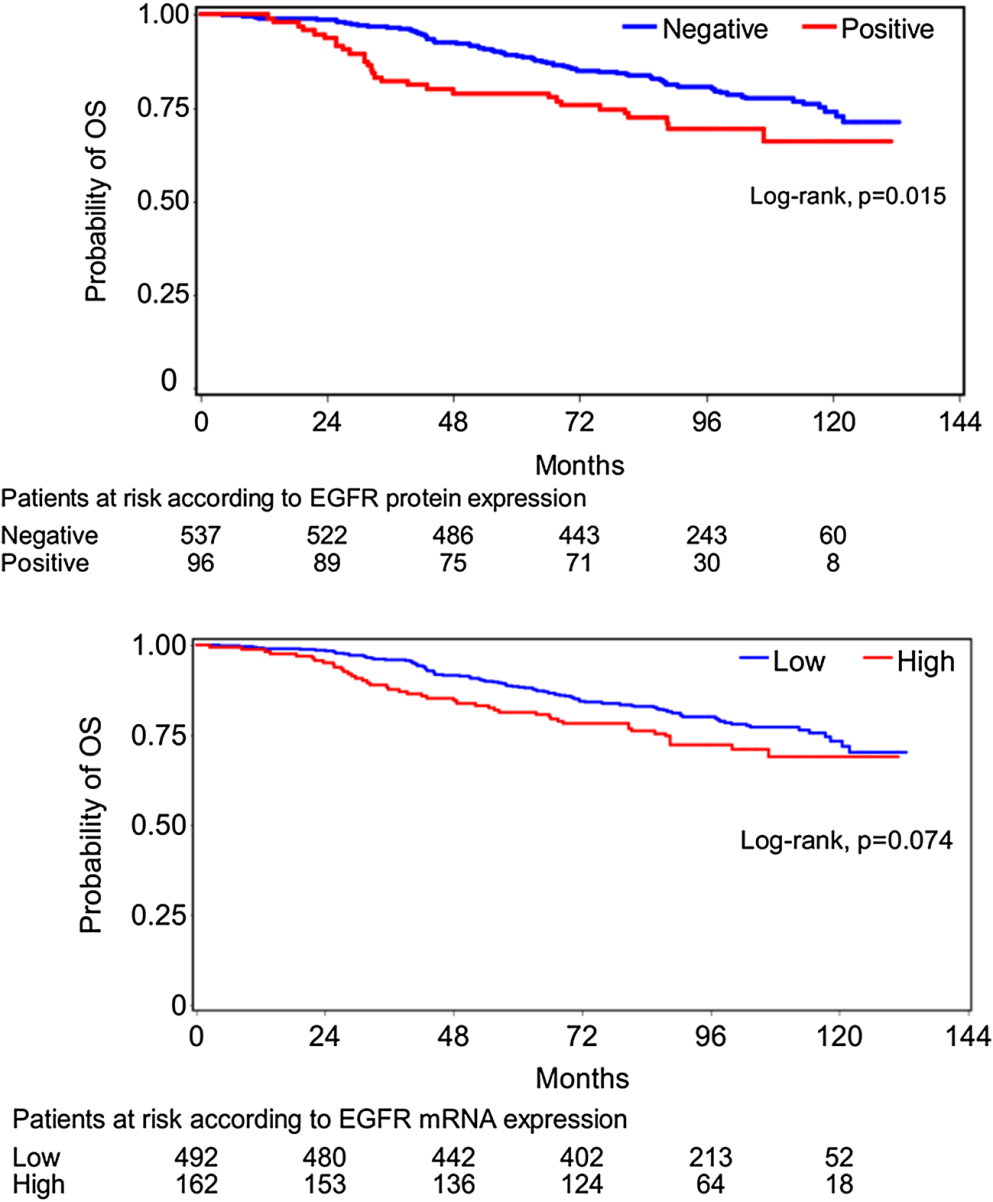
Overall survival according to EGFR protein and mRNA expression.

**Figure 5:**
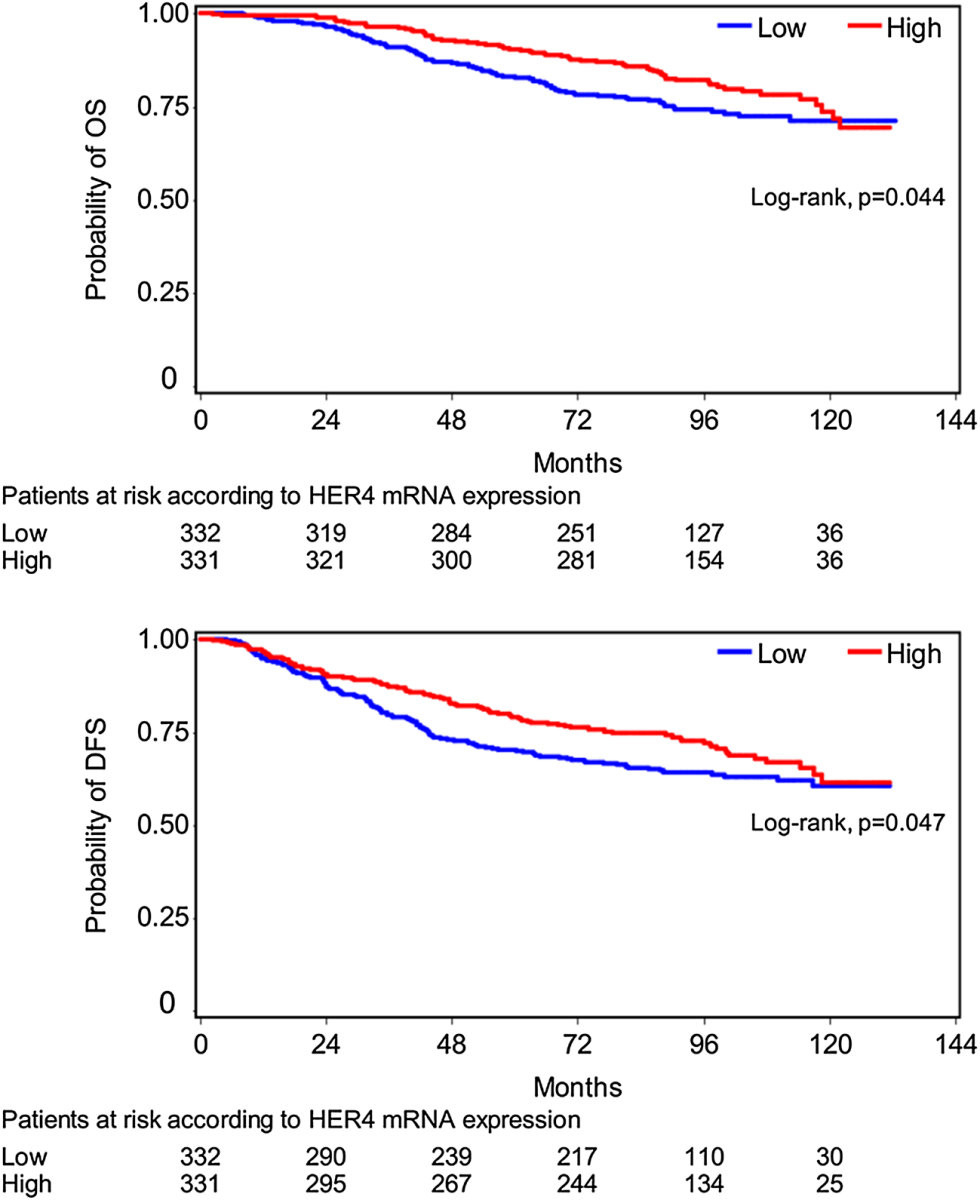
Overall survival and disease-free survival according to HER4 mRNA expression.

Concerning patients with high EGFR mRNA expression, a trend for significantly reduced OS (HR = 1.38, 95% CI 0.98–1.96, Wald’s p = 0.068, log-rank p = 0.074) was observed. However, with respect to protein expression, patients whose tumors were positive for EGFR had significantly reduced OS (HR = 1.65, 95% CI 1.10–2.48, Wald’s p = 0.017, log-rank p = 0.015) (Figure 4). No significant associations between HER2 (either mRNA expression or HER2 status by IHC/FISH) and OS were found. Similarly, HER3 mRNA expression was not associated with OS. In contrast, high HER4 mRNA expression (using the median value as a cut-off point) had a favorable prognostic value in terms of OS (HR = 0.72, 95% CI 0.52–0.99, Wald’s p = 0.045, log-rank p = 0.044) (Figure 5). In the multivariate analysis that included 612 patients, EGFR protein expression (HR = 1.66, 95% CI 1.06–2.60, Wald’s p = 0.028), tumor size (HR = 1.60, 95% CI 1.07–2.40, p = 0.023) and the number of positive nodes (HR = 2.74, 95% CI 1.86–4.04, p < 0.001) retained their prognostic significance for OS (Table 5).

**Table 5:** Cox regression multivariate analysis

Disease-free survival (N = 633)	Hazard ratio	95% CI	Wald’s p
Number of positive nodes			
≥4 vs. 1–3	2.13	1.58–2.87	**<0.001**
Type of surgery			
Mastectomy vs. breast-conserving surgery	1.49	1.07–2.06	**0.017**
Tumor size			
>2 vs. ≤2 cm	1.31	0.94–1.82	0.11
EGFR protein expression			
Positive vs. negative	1.56	1.09–2.24	**0.015**

Overall survival (N = 612)	Hazard ratio	95% CI	Wald’s p
Number of positive nodes			
≥4 vs. 1–3	2.74	1.86–4.04	**<0.001**
Type of surgery			
Mastectomy vs. breast-conserving surgery	1.48	0.98–2.24	0.066
Tumor size			
>2 vs. ≤2 cm	1.60	1.07–2.40	**0.023**
Adjuvant hormonal therapy			
Yes vs. no	0.70	0.48–1.04	0.074
EGFR protein expression			
Positive vs. negative	1.66	1.06–2.60	**0.028**

Missing data, regarding adjuvant hormonal therapy, resulted in a smaller number of patients included in the multivariate analysis for overall survival compared to disease-free survival.

Significant p values are shown in bold.

*CI* confidence interval.

With respect to DFS, no significant associations were demonstrated for EGFR, HER2 or HER3 mRNA expression. On the other hand, high HER4 mRNA expression was associated with lower risk for relapse (HR = 0.76, 95% CI 0.59–1.00, Wald’s p = 0.048, log-rank p = 0.047) (Figure 5). However, in multivariate analysis only EGFR protein expression (HR = 1.56, 95% CI 1.09–2.24, Wald’s p = 0.015), type of surgery (HR = 1.49, 95% CI 1.07–2.06, p = 0.017) and the number of positive nodes (HR = 2.13, 95% CI 1.58–2.87, p < 0.001) independently affected DFS (Table 5).

### Prognostic value of HER family receptor mRNA expression among subtypes

The prognostic significance of the HER family receptor mRNA expression among breast cancer subtypes was assessed by the use of univariate interaction tests. High EGFR mRNA expression was found to have unfavorable prognostic value in HER2-enriched cases in terms of OS (HR = 5.30, 95% CI 1.71–16.45, interaction p = 0.031). Adjusting for basic clinical and treatment characteristics, the prognostic ability of EGFR mRNA expression remained significant for OS, not only for the HER2-enriched tumors (HR = 9.12, 95% CI 2.87–29.03, Wald’s p = 0.0002), but also for the triple-negative patients (HR = 2.32, 95% CI 1.02–5.27, p = 0.045), with an overall interaction p value of 0.003 (data not shown).

### Prognostic value of HER family member co-expression

Regarding the prognostic significance of specific co-expression patterns of all four HER family receptors, we found that the combination of high EGFR, high HER2, low HER3, and low HER4 mRNA expression was associated with significantly worse DFS (log-rank, p = 0.033) and a trend for decreased OS (p = 0.065), compared with all other possible co-expression profiles.

## Discussion

In the current study, we used qRT-PCR to evaluate the transcriptional profiling of all four HER family receptor genes, in a large series of high-risk (predominantly T2-3, node-positive) early breast cancer patients, with a substantially long follow-up of 8 years. The aim of this analysis was to validate the findings of a previously published similar study, conducted from our group [7]. Most of the available studies have assessed protein expression and/or gene amplification of specific HER family members. Therefore, the prognosis of patients with breast cancer relating to the transcriptional profiling of all HER family receptors has not been extensively evaluated.

In accord with our previous results, the overall agreement between qRT-PCR (using the 50th percentile cut-off) and IHC/FISH for the determination of HER2 status was good (69%), with a Cohen’s kappa value of only 0.38, due to inherent differences in the cut-offs used in the two methods. These findings are in line with other studies [6, 22, 23]. Similarly, in agreement with our previous study the overall concordance between qRT-PCR (using the 75th percentile cut-off) and IHC for the evaluation of EGFR status was high (81%). Moreover, in complete agreement with our previous results, the qRT-PCR technique for HER2 (using the median value as a threshold) was associated with a high level of sensitivity (92%) and acceptable specificity (62%). The limitations regarding qRT-PCR as an alternative method for assessing HER family members in FFPE samples has previously been described [7].

Concerning the associations with clinicopathological parameters, our results are in compliance with our previous findings, as well. In general, HER3 and HER4 mRNA expression was associated with favorable, while EGFR and HER2 mRNA expression was associated with unfavorable parameters.

In the prognostic analyses, no significant associations between EGFR mRNA expression and OS were found in the total population, irrespectively of the cut-off points that we used. In our previous study [7], we demonstrated a reduced OS in patients with increased EGFR mRNA expression, using the 75th percentile as a cut-off. In the present analysis using the same cut-off, a trend (p = 0.074) towards a decreased OS was shown. Evaluating treatment arms separately, the difference in OS using the 75th percentile was significant in patients receiving the E–T–CMF regimen (p = 0.044). Moreover, EGFR mRNA expression was found to be a significant negative prognostic factor for OS in HER2-enriched and TNBC cases.

With regard to protein expression, patients with tumors positive for EGFR had significantly shorter OS in the total study population. Moreover, the negative prognostic significance of EGFR protein expression for OS and DFS was demonstrated in the multivariate analysis of the study. EGFR is considered to be a negative prognostic factor in breast cancer and such an association has been shown in ours, as well as in other studies [24–27].

In contrast to our previously reported data [7] and other studies [23, 28] no significant associations between HER2 status (either by mRNA expression or IHC/FISH) and OS or DFS were demonstrated. A definitive explanation for this unexpected finding cannot be given. Although adjuvant treatment with trastuzumab could be proposed to be a possible reason, none of the patients enrolled in the HE10/00 phase III trial received adjuvant therapy with trastuzumab. On the other hand, all patients had been treated with paclitaxel. So far, data concerning the interaction between HER2 receptor status and sensitivity to taxanes are not conclusive. However, the CALGB 9344 adjuvant study [29] has shown that patients with HER2-positive tumors had significant benefit from the addition of paclitaxel to doxorubicin/cyclophosphamide irrespectively of ER status, while there was no benefit in HER2-negative, ER-positive patients [30]. Moreover, a meta-analysis [31] of three trials [30, 32, 33] reported a significant interaction between HER2 status and adjuvant taxane therapy in terms of DFS. Such an interaction could possibly explain (at least in part) the absence of negative prognostic significance of HER2 status in the current analysis.

In our previous study [7] a positive association between HER3 mRNA expression and OS was reported. In contrast, no significant association between HER3 mRNA expression and clinical outcome was found in the present analysis. However, HER3 mRNA expression was associated with several favorable clinicopathological parameters such as ER and PgR positivity, histological grade I/II and fewer positive nodes. The available data in the literature regarding the prognostic role of HER3 in patients with breast cancer are contradictory [34]. Although a number of studies support a negative prognostic value of HER3 [25, 35, 36], other reports suggest a positive prognostic value [24, 37, 38]. So far, a conclusive explanation for the discrepancies among studies concerning the prognostic value of HER3 expression in breast cancer cannot be given. Recent data indicate that the sub-cellular distribution of HER receptors considerably affects their biological activities [39]. Therefore, the effect of HER3 on clinical outcome may be better evaluated taking into consideration, not only the expression of HER3, but also the sub-cellular distribution of the receptor and the expression levels of HER3 ligands [40].

In complete agreement with our previously reported results [7], an association of high HER4 mRNA expression (using the median value as a cut-off point) with increased OS and DFS was found. However, the positive prognostic value of high HER4 mRNA expression was not maintained in the multivariate analysis. Similarly, other studies have also demonstrated the positive prognostic ability of HER4 in patients with breast cancer, both at the mRNA and the protein level [24, 25, 41]. Existing evidence suggests that HER4 signaling promotes the differentiation and inhibition of growth in breast cancer cells [42]. In cell line experiments, when HER2-positive cancer cells were transfected to overexpress HER4, a reduction in proliferation and an increase in apoptosis was noted [43].

With regard to the prognostic potential of the combined expression patterns of the different HER family members, our findings indicate that it may be the co-expression patterns, rather than the expression of individual HER family receptors, that should be taken into account when assessing the prognosis of patients with breast cancer. This observation may prove to have important therapeutic implications.

## Conclusions

The present study suggests that EGFR protein overexpression, as assessed by IHC, is a negative prognostic factor for OS and DFS in patients with high-risk operable breast cancer. In addition, high HER4 mRNA expression was associated with a better clinical outcome in terms of OS and DFS in the univariate analysis. Since the clinical study was conducted in the pre-trastuzumab era, the above findings appear to have purely prognostic and not predictive significance. Furthermore, qRT-PCR may be a valid alternative technique for the determination of co-expression patterns of HER family receptors in FFPE breast tumor samples with possible important therapeutic implications.

## Abbreviations

CMF: cyclophosphamide/methotrexate/5-fluorouracil
CT: cycle threshold
DFS: disease-free survival
DNA: deoxyribonucleic acid
E: epirubicin
EGFR: epidermal growth factor receptor
ER: estrogen receptor
FFPE: formalin-fixed paraffin-embedded
FISH: fluorescence in situ hybridization
HECOG: Hellenic Cooperative Oncology Group
HER: human epidermal growth factor receptor
IHC: immunohistochemistry
mRNA: messenger RNA
OS: overall survival
PCR: polymerase chain reaction
PgR: progesterone receptor
qRT-PCR: quantitative reverse transcription-polymerase chain reaction
RQ: relative quantification
RNA: ribonucleic acid
T: paclitaxel
TMA: tissue microarray
